# The Institutional Library

**Published:** 1916-05-06

**Authors:** 


### THE INSTITUTIONAL LIBRARY.

**How to Live Long.** By J. Walter Carr, M.D., F.R.C.P. (London: Methuen and Co., Ltd. Pp. 114 + viii. Price 1s. net.)

This is one of a series of popular handbooks which profess to deal with the positive side of health in non-technical terms and for the lay reader. The object of the scheme will commend itself generally to the medical profession, and all the more so that these volumes make no attempt to rival such productions as "The Household Physician" or "Every Man his Own Doctor." That men and women should have an intelligent appreciation of the laws which govern personal health must be the desire of every medical practitioner, for such knowledge is a help to national well-being as well as a factor making for a due comprehension of the aim of medicine. The volume written by Dr. Walter Carr has a title which is sure to make a wide appeal, and most practitioners would agree with the majority of his counsels. These are distinguished by moderation and common sense, and on such subjects as food, alcohol, exercise, etc., he keeps closely to practical issues, and is not led away into any extremes or fads. The question of limitation of families is a delicate one, and though what Dr. Carr says on this point seems to us eminently reasonable, we are not sure but that he may get into trouble in other directions. On the whole he has performed successfully a task which is not without its difficulties.

**Health for Young and Old: Its Principles and Practice.** By A. T. Schofield, M.D. Brux. (London: William Rider and Son, Ltd. 1916. Price 2s.)

It is impossible entirely to take this book seriously. The author is obviously proud of the unconventional character of his manual, and on many a page a straining after this attribute is apparent. This factor does not, however, enhance the value of his book as a guide to health. In the course of its 260 pages there are a great many bits of advice thoroughly to be recommended and not a few little lectures which should be taken to heart. But it may be feared that the lay reader will be unable to distinguish clearly the wheat from the chaff. Some of the inaccuracies are unimportant from his point of view, such as the description of the function of the epiglottis. What is the point in saying that the water supply of the Metropolis is worse than that of any English city, and in quoting a thirty-year-old authority to the effect that "out of every ten glasses of London water one had been drunk before"? One would hardly expect a medical man to suggest that rubber-heels would greatly lessen brain-jars. An example of rash statement is the pronouncement that the index-finger in women is generally longer than the middle one. Some chapters in this discursive manual are at least entertaining, and passable witticisms are not infrequent, but as a manual of hygiene it will not be a success until it has been overhauled.

### Institutional Library—*continued from p. 130.*

**Modern Medicine and Some Modern Remedies.** By THOMAS BODLEY SCOTT. With a Preface by Sir LAUDER BRUNTON, Bart., F.R.S. (London: H. K. Lewis and Co., Ltd. 1916. Pp. 159 + xv. Price 4s. 6d. net.)

WITH the motive which has led to the writing of this book everyone interested in practical medicine must necessarily sympathise. It is a desire to record and to make available to others the lessons which have been learned by the author in general practice. We quite agree with Sir Lauder Brunton's contention that much is lost because general practitioners contribute so rarely to the columns of our journals and to the medical Press. But when, impressed by this argument, we turn to Dr. Scott's pages we are obliged to confess a severe disappointment. Not unnaturally we had hoped to learn the personal convictions of the author on the practical problems of medicine, the reasons for these convictions, and the therapeutic methods to which they conducted. But of clear, decided, personal clinical teaching, whether empirical or rational, there is but scanty measure, and only too often the problems which are stated are left without an answer. Take, for example, the influence of digitalis on blood-pressure. On this subject there have been a number of hospital records which show that, contrary to the general teaching, digitalis does not cause a rise of blood-pressure. Dr. Scott recognises that the problem is an important one as bearing on treatment. But all he does to solve it is to "think strongly" that were observations made on non-hospital patients a different result would be obtained. If he had made these observations and recorded them he would have made a useful contribution to medical practice, but mere thinking, "strongly" or otherwise, on such a matter is a vain thing. To put our point in general terms, our complaint is that the book gives us far too little of Dr. Scott's first-hand knowledge and clinical teaching and far too much of what someone else has thought, or suggested, or believed, or imagined. In a volume which offers itself as one of practical lessons from general practice superficial summaries of well-known authors and long quotations from numerous authorities are entirely out of place; but in Dr. Scott's pages they abound. We regret to find ourselves unable to accept Sir Lauder Brunton's conclusion that the multiplication of books such as the one now before us would be a great gain to the medical profession. That it contains some few points worthy of note we readily admit, but the diluting material is in excess, and the note of first-hand knowledge sounds but faintly. It is, in short, weak in the very direction in which its profession entitles us to expect it to be strong; and, to complete the indictment, it has no index.

**Nervous Asthma: Its Pathology and Treatment.** By J. B. BERKART, M.D. (London: Humphrey Milford, Oxford University Press. 1915. Pp. 54. Price 2s. 6d. net.)

IN his position as physician to the City of London Hospital for Diseases of the Chest, Dr. Berkart has had special opportunities for the study of respiratory affections, and in this small volume he discusses the subject of asthma on the basis of a large personal experience. He recognises the frequency with which other forms of dyspnœa are confounded with the true nervous variety, and insists that in this latter there does not exist any mechanical obstacle in the air-passages or any interference with the ingress and egress of air to and from the lungs. There may be a complicating bronchial catarrh, but the products of this are easily expectorated and do not block the tubes, and in this respect the true nervous asthma differs from the bronchial variety, where a coagulated fibrinous exudation is formed in the air-passages and is expelled with difficulty. He does not accept the widely alleged statement that in the asthmatic paroxysm there is a constriction of the muscles of the bronchial tubes. His view, in brief, is that the asthmatic patient is one who is given, usually by inheritance, a nervous system prone to functional disturbances; that in early life the patient has suffered from some inflammatory affection of the air-passages; that, in view of the family history, the dyspnœa which accompanies these affections is named "asthma"; and that under these influences there is inflicted a "psychic trauma" which renders the respiratory process easily subject to mental or other influences. The paroxysm itself is an anxiety neurosis excited by the fear of an impending dyspnœa. Such a view has necessarily an important bearing on treatment, and this is dealt with very fully and with marked soundness of judgment. Dr. Berkart's essay is worthy of very careful study, and his practical advice is both helpful and authoritative.

**School Children: Their Care and Nursing.** By ISABEL MACDONALD, D.N., F.I.H., A.R.S.I. (London: The Scientific Press, Ltd. 1s. net. Pocket Guide Series.)

THIS small handbook creates a favourable impression at the outset by its light, compact form, delightfully clear, simple style, and the eminently practical nature of its contents. As a result of years of experience the writer has realised fully, from the school nurse's standpoint, the differences between children as found in schools and the children hitherto under her care in hospital or private nursing. For lack of the specialised information here so concisely supplied the most highly-trained woman may well find herself at a loss on first taking up this branch of public health work. Nearly the whole range of disease as seen under school conditions is passed under review, the chapters on first aid, skin ailments, and infectious diseases being particularly good and full of practical hints. The effects of mental fatigue, anæmia, and heart disease are excellently described, and much sound advice is given on the hygiene and management of children affected.

In dealing with tests for hearing the writer would add to the usefulness of her book if she described the "forced whisper" test enjoined by the Board of Education. Her accounts of the origins and causes of disease contain occasional slips, but this in no way detracts from a manual which should prove of the greatest value to an increasingly numerous and important body of nurses.

**The Eyes of Our Children.** By N. BISHOP HARMAN, M.A., M.B. Cantab., F.R.C.S. Eng. (London: Methuen and Co., Ltd. Pp. 119. 1s. net.)

THIS little book is one of Messrs. Methuen's "Health Series," of which each volume is by a separate author, and treats of a special medical subject. These works are intended for the instruction of the lay public, their very moderate price bringing them within reach of all. As to the advisability or otherwise of the publication of such a series medical opinions doubtless differ, but in forming a judgment of an isolated unit, such as the book under consideration, the effect likely to be produced by its perusal on the readers for whom it is intended must, before all things, be taken into consideration. From this point of view the subject-matter may be divided into two portions, which are, indeed, largely interwoven, but each of which roughly occupies about half the book. These are (*a*) the portion which consists of elementary embryology, anatomy, normal and morbid, together with a certain amount of physiology; and (*b*) the portion which deals with advice on various subjects, such as headache, myopia, squint, lighting, reading, and the like. As regards the portion which we have described under (*a*), we are inclined to doubt whether it will serve any very useful purpose. The (in medical matters) uninstructed public are notoriously prone to "seize the wrong end of the stick" when reading anything in the nature of popular pathology, and thus to form erroneous but firmly fixed ideas, which not infrequently prove decidedly embarrassing to those medical practitioners whose lot it is to deal with persons who have formed them. With regard, however, to the parts of the book which we have grouped under (*b*), they contain much excellent advice which will doubtless prove of great benefit to the children of those parents who act upon it.

**Cerebro-Spinal Fever.** By Michael Foster, M.D., and J. F. Gaskell, M.D. (Cambridge University Press. 1916. Pp. 222. Price 12s. 6d. net.)

There is plenty that is notable in this monograph besides the high technical excellence of its *format* and the conjunction of names on its title-page so redolent of the Cambridge Scientific School. Fortunately, Drs. Foster and Gaskell have no need to rely upon the prestige of their celebrated parents. Their account of the disease known in this country as cerebro-spinal fever is not merely the most thorough and complete published in England, but has the additional advantage that it represents the clinical and pathological experiences of two men who devoted themselves last year to the study of (part of) the epidemic which occurred in the Home Army. It is beautifully illustrated with coloured and other plates, and deals fully with every aspect of the disease. A copious bibliography is another point deserving commendation. We note that the authors, agreeing in this with most other clinicians in this country, have been disappointed with the serum treatment, and much prefer simple lumbar puncture, repeated as often as necessary. They disagree with other authorities, however, as to the frequency of tonsillar and pharyngeal symptoms at the onset of the disease: evidently they met with cases in which these symptoms were mainly absent or overlooked, but the experience of most other authorities (and of the present reviewer) is that such symptoms are exceedingly common. The book as a whole is admirable, and reflects great credit on all concerned in its production: incidentally, the price is exceedingly moderate for so sumptuously produced a volume.

**Cleft-Palate and Hare-Lip.** By Sir W. Arbuthnot Lane, Bart., M.S., F.R.C.S. Third Edition. 1916. (London: Adlard and Sons.)

For a good many years now Sir W. Arbuthnot Lane has been advocating principles of operative treatment for these congenital deformities which are quite opposed to those accepted by most other surgeons. In spite of his ingenuity and industry, and in spite of the prestige which he has derived from pioneer work in other branches of surgery, he has not convinced his colleagues that he is right in these matters; indeed, the general verdict is that he is hopelessly and entirely wrong. That this opinion is not based upon mere conservatism, mere inability to abandon a tradition, may be safely deduced from the universal acceptance of Sir W. A. Lane's views upon certain other subjects—for instance, the operative treatment of fractures. Like the leader of men that he is, he sticks nevertheless to his own opinions, and in this third edition of his book reiterates his beliefs. But as a piece of argument the monograph lacks cogency for several reasons. First, too little account or too cavalier account is taken of divergent views, such as those so ably upheld by Mr. Berry and Mr. Goyder, to mention only two of those with whom he has been in controversy. Secondly, the first thirty pages are, if not actually irrelevant, at any rate of secondary importance. Thirdly, the composition is slovenly in many places, and the arrangement of the material poor. Sir Arbuthnot will have to put his pleadings into better shape before he is likely to get the verdict on this question reversed; at least he can go on appealing as long as he likes, for there is no final or unalterable judgment in surgery.

**The Care of the Body.** By Francis Cavanagh. Fcp. 8vo. Pp. 151. Methuen. 1s. net.

In this invaluable primer of health and fitness the whole routine of life is passed in review, and advice is given on all points towards gaining and conserving strength. We know this is so, for we have it on the very best authority, that of the author or the publisher himself, who prints this unbiassed opinion, with other laudatory matter, on the paper in which the book is wrapped. A hundred and fifty pages seem a good deal to devote to instructing the ordinary man, woman, and child how to sleep, bathe, take exercise, wear clothes, care for his skin, teeth, feet and hands, eyes, ears, nose, and, generally, how to preserve his health and live long; but evidently it is not enough, for in the same series there are separate books, presumably of the same size, on respectively Health for the Middle Aged, Throat and Ear Troubles, Care of the Teeth, of the Eyes of Children, of the Skin, on the Prevention of the Common Cold, and on How to Live Long. Evidently the series is as yet incomplete. It is very well and very excellent for the middle aged to be taught how to preserve health, but we trust they are not to have a monopoly of this useful knowledge. We look for other volumes on Health for the Baby, Health for the Schoolboy, Health for the Schoolgirl, Health for the Adolescent, Health for the Debutante, Health for the Traveller, Health for the Sedentary, Health for the Old; and when these are completed we look for a series of Health for the Parson, Health for the Doctor, Health for the Sailor, the Soldier, the Blacksmith, and persons of other occupations. Moreover, we should like to see other books treating on Health for the Tall, Health for the Short, Health for the Fair, Health for the Dark, and so on. It is an admirable thing that everyone should be instructed how to care for his eyes, his teeth, his skin, his throat, and his ears, but why not also his tongue, his stomach, his fingers and toes, his lungs, and, above all, his liver? The common cold is no doubt a severe infliction, and we are grateful to Dr. O. K. Williamson for telling us how to prevent it, but what is the suffering inflicted by a common cold in comparison with that due to a chill on the liver? A book on this subject would have an enormous sale, and we commend the notion to the enterprising publisher of this series. By the sample under notice we should say that the series is well adapted to educate the readers into confirmed valetudinarians.

